# Latent dimension linking functional connectivity with post-stroke deficits across multiple domains

**DOI:** 10.1093/braincomms/fcaf276

**Published:** 2025-07-22

**Authors:** Ke Wu, Yaya Jiang, Junhao Luo, Yijun Chen, Shaoling Peng, Xiangyu Kong, Gaolang Gong

**Affiliations:** State Key Laboratory of Cognitive Neuroscience and Learning & IDG/McGovern Institute for Brain Research, Beijing Normal University, Beijing 100875, China; Artificial Intelligence and Language Cognition Laboratory, Beijing International Studies University, Beijing 100024, China; State Key Laboratory of Cognitive Neuroscience and Learning & IDG/McGovern Institute for Brain Research, Beijing Normal University, Beijing 100875, China; Shenzhen CyberAray Network Technology Co., Ltd, Shenzhen 518038, China; Harbin Institute of Technology (Shenzhen), Shenzhen 518055, China; Department of Psychology, Georgetown University, Washington, DC 20057, USA; Department of Neurology, Boston Children’s Hospital, Harvard Medical School, Boston, MA 02115, USA; State Key Laboratory of Cognitive Neuroscience and Learning & IDG/McGovern Institute for Brain Research, Beijing Normal University, Beijing 100875, China; State Key Laboratory of Cognitive Neuroscience and Learning & IDG/McGovern Institute for Brain Research, Beijing Normal University, Beijing 100875, China; Beijing Key Laboratory of Brain Imaging and Connectomics, Beijing Normal University, Beijing 100875, China; Chinese Institute for Brain Research, Beijing 102206, China

**Keywords:** stroke, functional connectivity, recovery, lesion, structural disconnection

## Abstract

Stroke often leads to multiple behavioural impairments. Understanding the neural basis of these deficits is essential for elucidating the mechanisms of functional impairments and optimising therapeutic strategies for stroke patients. Although many studies have revealed that specific behavioural deficits are related to disruptions in distributed functional connectivity across brain networks, these studies typically focus on single behavioural traits, overlooking the multivariate characteristics of deficits after stroke. Recent studies have demonstrated that deficits within and across domains are highly correlated, suggesting a complex many-to-many mapping between brain and behaviour following stroke. Thus, the present study aims to identify meaningful multivariate patterns of functional connectivity–behaviour covariation following stroke. Specifically, we employed a multivariate data-driven approach, partial least squares correlation, to examine the relationships between whole-brain functional connectivity and an extensive array of neurological scores (including motor, attention, verbal memory, spatial memory and language domains) in a large cohort of stroke patients at 2 weeks (*n* = 81), 3 months (*n* = 78) and 12 months (*n* = 74) post-injury. This multivariate analysis revealed a significant latent component (LC) from 2-week post-stroke data, capturing a unique pattern of cognitive deficits across multiple domains. This pattern was strongly associated with widespread network dysfunction, characterized by decreased interhemispheric connectivity and increased intrahemispheric connectivity. Notably, the identified LC was replicated and generalized to stroke data at the 3-month and 12-month time points. Furthermore, we examined whether structural lesion features, including structural disconnection of white matter pathways and grey matter damage, could explain variance in the identified LC. Structural disconnection outperformed grey matter damage, highlighting its critical role in the functional connectivity–behaviour relationship following stroke. Mediation analysis confirmed that structural disconnection serves as the neuroanatomical basis for the association between functional connectivity and deficits. Overall, this study suggests that stroke-induced white matter disconnections are associated with widespread and consistent disruptions in brain network connectivity, which are reflected in a highly correlated behavioural profile. These results provide an integrative insight into the complex relationships among lesions, functional networks, and behavioural outcomes following stroke.

## Teaser

Ke *et al*. explored the associations between multi-domain deficits and whole-brain functional connectivity in stroke patients. A maintained functional connectivity-deficit covariance pattern exists from acute to chronic stages of recovery, with white matter disconnections identified as the key structural basis.

## Graphical Abstract

**Graphical Abstract:**
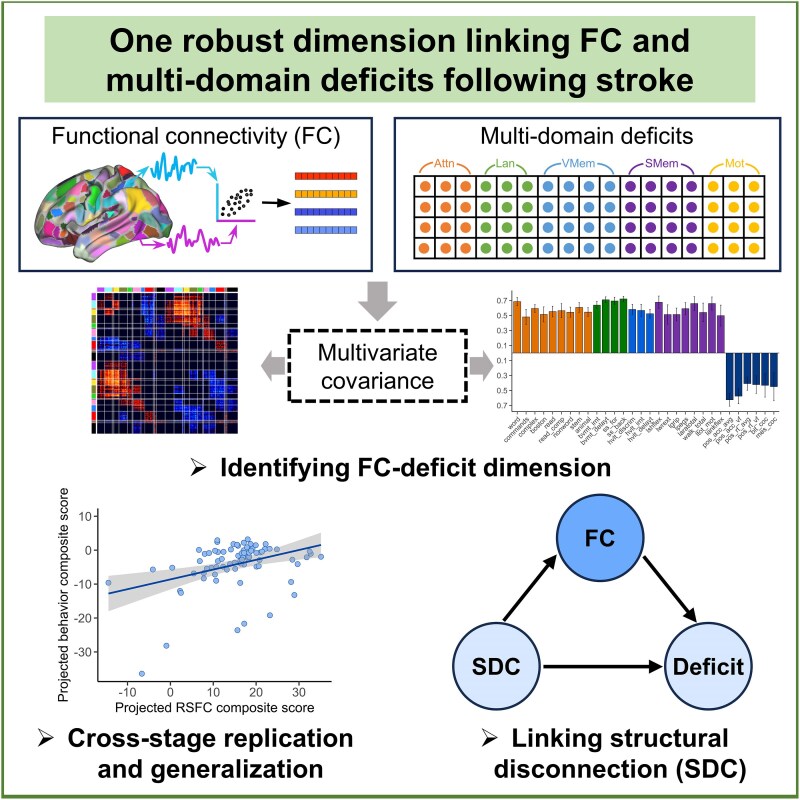

## Introduction

Stroke is one of the leading causes of disability and death worldwide. Stroke patients generally suffer from behavioural deficits in multiple domains, e.g. motor, language, attention, and executive functions.^1-4^ The occurrence of multiple deficits reflects not merely structural damage at the injury site,^5,6^ but also widespread dysfunction within and between networks.^7-11^ Elucidating the associations between brain functional networks and behavioural performance across multiple domains in stroke patients is crucial for understanding the mechanisms of neurological disorders and improving the treatment strategies.^12,13^

Functional magnetic resonance imaging (fMRI) during the performance of specific tasks, e.g. motor, language, and attention, has been widely applied in the study of stroke patients, shedding light on functional abnormalities or reorganisation following stroke.^11,14,15^ However, this technique has certain limitations when used in stroke studies. First, task-based fMRI studies often exclude stroke patients with severe functional deficits due to their inability to complete experimental tasks designed for healthy individuals.^14^ This selection bias may limit the generalizability of findings to the broader stroke population. Moreover, activation paradigms refer to task-based designs that probe specific functional systems, but are typically optimized for intact neural circuits rather than reorganized systems following stroke.^16,17^ Additionally, task-evoked studies provide limited information about temporal interactions between regions or networks.^16^ Resting-state functional MRI has emerged as a powerful tool to map the functional network connectivity of damaged brains by measuring the temporal correlation of blood oxygen level-dependent (BOLD) signals across brain regions at rest [resting-state functional connectivity (RSFC)].^18,19^ Studies have demonstrated that severity of neglect symptoms in hemispatial neglect patients is correlated with FC alteration within and between (i.e. interactions) multiple resting-state networks other than specific attention networks.^8,20,21^ A subsequent study explored FC–deficit relationships across six behavioural domains (attention, spatial memory, verbal memory, language, motor and visual) in a large, heterogeneous cohort of stroke patients and revealed that each deficit was predicted by a common RSFC pattern characterized by decreased interhemispheric connectivity and increased intrahemispheric connectivity.^22^ Together, these lines of study indicate that RSFC offers crucial insights into the functional network organisation associated with different domains of behavioural performance after stroke.

Notably, most resting-state fMRI studies have used univariate correlation or multivariate regression approaches to highlight that behavioural dysfunction in a specific domain or task is related to FC disruptions across multiple networks, but they often neglected the potential correlations between behavioural dysfunctions across multiple domains or tasks and FC disruptions across multiple networks.^8,20-25^ Evidence at the behavioural level has demonstrated that focal and diffuse strokes do not typically cause highly specific and distinct syndromes or symptoms but instead result in deficits that are closely correlated both within and between behavioural domains at the population level.^23,24,26^ Traditional explanations for this phenomenon suggest that strokes occur within vascular territories involving multiple functional areas.^12^But correlated deficits have also been observed between regions without obvious vascular overlap. For example, language and spatial memory deficits are significantly correlated (*r* = 0.59), despite being localized to different hemispheres.^23^ Another plausible explanation is the correlation of multiple deficits coupled with widespread and consistent disruption in network connectivity, as evidenced by the interdependent recovery of deficits across multiple domains.^12,13,24^ This argument supports many-to-many mapping between behavioural deficits and functional network connectivity after stroke. However, until recently, few neuroimaging studies have simultaneously investigated both the multivariate characteristics of behavioural deficits and the multivariate features of functional network connectivity to identify meaningful patterns of FC-behaviour covariation after stroke.

In this study, we employed partial least squares correlation (PLSC) analysis, a multivariate data-driven approach, to examine the relationships between whole-brain FC and an extensive array of neurological assessment scores. This analysis allows multivariate brain features to be mapped to multivariate behavioural variables in a single integrated analysis, thereby avoiding the pitfall of focusing on a single a priori behavioural aspect, which hides the overall picture.^27-32^ Additionally, we examined whether the latent component (LC) identified in the 2-week stroke data remained stable throughout the recovery process. Previous studies have suggested the recovery of post-stroke deficits dependent on the normalisation of abnormal network connectivity.^21^ On this basis, we hypothesized that the behaviour–connectivity covariance patterns would be maintained from the acute to the chronic stage. Furthermore, post-stroke structural brain lesions, including grey matter damage or white matter disconnection, are closely associated with widespread FC abnormalities and multiple behavioural deficits.^33-35^ However, few studies have explored the complete relationship linking structural damage to widespread FC disruptions and multidomain behavioural deficits. To address this gap, we conducted mediation analyses to deepen the understanding of the relationships among these three variables.

## Materials and methods

### Dataset

The data used in this study are part of the Washington Stroke Cohort.^22-24^ The dataset includes 132 patients with a first symptomatic stroke, who were assessed at 2 weeks after stroke onset. Of these, 103 and 88 returned for the subsequent measurements at 3 and 12 months, respectively. A healthy control (HC) group (*n* = 31), matched with the study sample for age, gender and years of education, was assessed twice at 3-month intervals. Written informed consent was obtained from all participants in accordance with the Declaration of Helsinki and procedures established by the Washington University in Saint Louis Institutional Review Board. All aspects of this study were approved by the Washington University School of Medicine Internal Review Board.

The inclusion criteria of present study were as follows: ischaemic or haemorrhagic stroke, and clinical evidence of motor, language, attention, visual, or memory deficits based on neurological examination. The exclusion criteria were as follows: (i) participants with data missing from more than one behavioural domain; (ii) bilateral lesions; and (iii) poor-quality T1-weighted images and fMRI data. Finally, 81 patients were eligible at 2 weeks, 78 at 3 months, and 76 at 12 months; 28 and 26 controls were eligible at the first and second assessments, respectively (Table 1).

**Table 1:** Demographic data of participants in the present study

	Stroke patients			Control healthy	
	2 weeks	3 months	12 months	Visit 1	Visit 2
**Number**	81	78	74	28	26
**Age**	52.2 ± 11.4	53.8 ± 10.6	54.1 ± 9.7	55.1 ± 12.3	55.7 ± 11.8
**Gender (M/F)**	48/33	45/33	43/31	13/15	16/12
**Education (years)**	13.4 ± 2.6	13.3 ± 2.4	13.6 ± 2.5	13.4 ± 2.6	13.5 ± 11.8
**Handedness (R/L)**	73/8	72/6	68/6	27/1	24/2
**Lesion type (ischaemic/haemorrhagic)**	68/13	56/14	64/10		
**Lesion side (R/L)**	38/43	35/43	34/40		
**Lesion size (cm^3^)**	3.29 ± 5.27	3.2 ± 4.6	2.8 ± 3.5		

### Neuropsychological assessment

The neuropsychological battery comprised 41 behavioural scores across five behavioural domains: language, attention, verbal memory, spatial memory, and motor function. Detailed descriptions of the behavioural tests are available in the Supplementary Table 1. To determine whether the missing data were missing completely at random (MCAR) or not, we conducted Little's MCAR test.^36^ The test yielded a statistically significant result (*P* < 0.001), indicating that the missing data were not completely random. In this context, we adopted a mean imputation strategy that preserves as much information as possible while maintaining the integrity of the analysis.

### Neuroimaging data collection

Images across various modalities, including structural, functional, pulsed arterial spin labelling (PASL), and diffusion tensor scans, were acquired using a Siemens 3T Tim-Trio scanner with a 12-channel head coil. Structural scans included sagittal T1-weighted MP-RAGE [repetition time (TR) = 1950 msec, echo time (TE) = 2.26 msec, flip angle = 90°, voxel size = 1.0× 1.0× 1.0 mm], transverse T2-weighted turbo spin echo (TR = 2500 msec, TE = 435 msec, voxel size = 1.0× 1.0× 1.0 mm) and sagittal fluid attenuated inversion recovery (FLAIR) (TR = 7500 msec, TE = 326 msec, voxel size = 1.5× 1.5× 1.5 mm). The PASL acquisition parameters were as follows: TR = 2600 msec, TE = 13 msec, flip angle = 90°, bandwidth = 2.232 kHz/Px, and field of view (FoV) = 220 mm; 120 volumes were acquired (322 s total), each containing 15 slices with slice thicknesses of 6 mm and gaps of 23.7 mm. Resting-state functional scans were acquired with a gradient echo planar imaging sequence (TR = 2000 msec, TE = 27 msec, 32 contiguous 4-mm slices, 4× 4 mm in-plane resolution). The participants fixated on a small cross in a low-luminance environment. A total of six to eight fMRI scans, each consisting of 128 volumes, were collected over approximately 30 min.

### Lesion identification

Lesions were manually segmented on individual structural MR images with the Analyze biomedical imaging software system.^37^ The T1-weighted, T2-weighted, and T2-FLAIR scans were used together to ensure accurate lesion delineation. Two board-certified neurologists reviewed all the segmentations, and an additional neurologist confirmed all the segmentations. For haemorrhagic strokes, oedema was included in the lesion. The final segmentations were used as binary lesion masks for subsequent processing and analysis steps. Lesion masks were transformed to Montreal Neurological Institute atlas space via a combination of linear transformations and non-linear warps and were resampled to achieve isotropic voxel resolution.

### fMRI data preprocessing

Resting-state fMRI preprocessing consisted of (i) removal of the first four frames and brain extraction; (ii) compensation for asynchronous slice acquisition via sinc interpolation; (iii) whole-brain intensity normalisation to achieve a mode value of 1000; (iv) spatial realignment within and across functional MRI runs; and (v) cross-modal image registration and normalisation. In the fifth step, functional MRI volumes from each time point were transformed from native space to standard space via deformation fields obtained from our previous longitudinal voxel-based morphometry analyses.^38^

In preparation for the FC analysis, data underwent several additional preprocessing steps: (i) spatial smoothing consisting of a 6 mm full-width at half-maximum Gaussian blur in each direction; (ii) temporal filtering retaining frequencies in the 0.009–0.08 Hz band; (iii) regression of head motion, signals from ventricles and cerebrospinal fluid, signals from white matter, and the global signal; (iv) scrubbing using the DVARS measure; and (v) removal of the mean signal intensity over the run. Notably, the calculation of the brain signal in the third step did not consider the signal of the lesion location.

### FC features

We used the Gordon 333 cortical parcellation and network community assignments to obtain region-level and network-level measures of FC. This parcellation was based on FC boundary mapping and InfoMap community detection analyses of resting-state fMRI data from 120 healthy individuals.^39^ A total of 333 regions of interest (ROIs) assigned to 13 large-scale functional networks were used for subsequent estimation of FC. Region-wise FC matrices were constructed by correlating the average nuisance-regressed BOLD time series of each region with the average nuisance-regressed BOLD time series of every other region and applying the Fisher *z*-transformation to the resulting linear correlation values. For each patient, voxels that fell within the boundaries of the lesion were masked out, and regions with more than 50% lesion overlap were excluded from all analyses, in accordance with previous reports.^40^

### Structural lesion features

Structural lesion features included region-based damage and region-based structural disconnection (SDC). Region-based damage was calculated as the percentage of voxels within grey matter regions overlapping with the lesion, creating a 1-dimensional vector. This measure quantifies the damage to predefined brain regions rather than individual voxels. Region-based SDC was defined as the proportion of streamlines connecting two regions that were disrupted by the lesion, which was determined with the Lesion Quantification Toolkit (LQT). The LQT incorporates a publicly available diffusion MRI streamline tractography atlas to create a structural connectome template.^41^ On the basis of 333 cortical parcellation inputs and the Human Connectome Project-842 tractography atlas, a 333×333 region-wise structural connectome was created. By embedding the lesion into the atlas-derived structural connectome, the LQT generated a 333×333 SDC matrix where each value represents the severity of lesion-induced disruption in structural connectivity between two brain parcels. Before conducting the statistical analyses, the upper triangles (excluding diagonal elements) of the disconnection matrices were extracted and reshaped into a 1-dimensional vector.

### Statistical analysis

#### PLSC analysis

**Figure 1:**
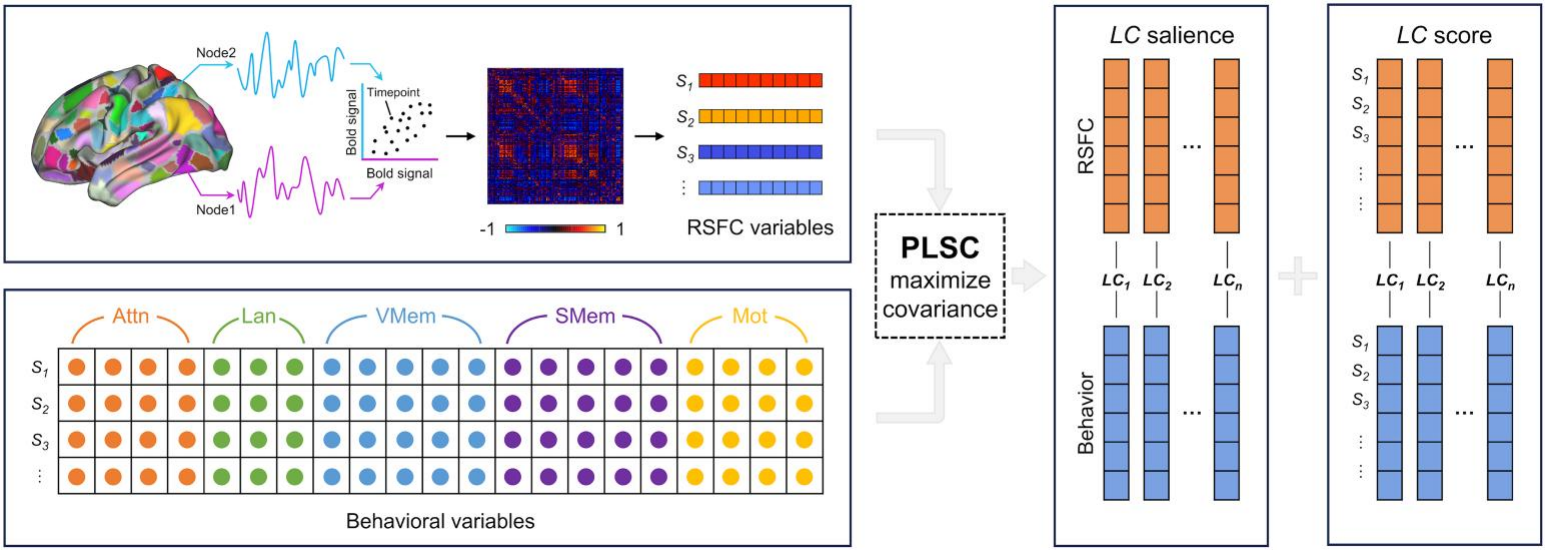
**Diagram of PLSC.** In the context of exploring brain–behaviour associations, the inputs to the PLSC model consist of a whole-brain FC matrix and a behaviour matrix. For brain inputs, BOLD signal time series are extracted from predefined cortical parcellations. A whole-brain FC matrix is constructed for each subject, and the upper triangles (excluding diagonal elements) of this matrix is reshaped into a one-dimensional vector. For behaviour inputs, item-level data for each subject, including various assessments spanning the motor, language, attention, verbal memory and spatial memory domains, are incorporated into the PLSC model. The model identifies FC saliences and behavioural saliences, which represent linear combinations of the variables in the FC and behaviour matrices. By projecting the original FC and behaviour data onto their respective saliences, individual FC and behaviour composite scores are derived. The model optimized these saliences to maximize the covariance between the brain composite scores and the behavioural composite scores.

We used PLSC analysis to examine the relationships between whole-brain FC and multiple neurological measures across five behavioural domains (Fig. 1). Both sets of variables were correlated with each other across participants, and the resulting covariance matrix was subjected to singular value decomposition to reveal the latent brain–behaviour components. Each LC comprised an RSFC pattern at the node level (RSFC saliences) and a profile of behavioural deficits (behavioural saliences). Individual-specific RSFC and behavioural composite scores for each LC were obtained by linearly projecting the original RSFC and behavioural data of each participant onto their respective saliences.

We computed Pearson's correlations between participants’ composite scores and the original data, referred to as loadings, to interpret the contribution of each variable to the LCs.^27,42^ A strong positive or negative correlation for a specific behavioural measure in a given LC indicates the greater significance of that behavioural measure to the LC. Likewise, a large positive (or negative) correlation for a specific RSFC measure in a given LC highlights the importance of the RSFC measure.

A permutation test (1000 permutations) was used to determine the significance of each individual LC.^43^ Additionally, bootstrap resampling (1000 bootstraps) was employed to estimate CIs of the behavioural and RSFC loadings. Procrustes rotation was applied to the LCs obtained from the permutation and bootstrap analyses to ensure alignment with those from the original analyses.^43,44^ Bootstrapped *z* scores were calculated by dividing each loading by its bootstrap standard deviation, and then the *z* scores were converted to *P* values. Prior to this, bootstrapped RSFC loadings were averaged across ROI pairs within and between 26 networks (left: 13; right: 13), resulting in 26× 26 correlation matrices to limit the number of multiple comparisons.

We also used PLSC analysis to examine the relationships between region-based SDCs and multiple-domain neurological variables. For further details, refer to the above analysis. To visualize the SDC loadings, the top 20% of loadings for LC1 were mapped onto the brain to display the most predictive SDCs using BrainNet Viewer. To assess whether LC1 from the SDC–deficits PLSC model corresponds to LC1 from the FC–deficits PLSC model, we calculated the Pearson correlation coefficient between the LC1 behavioural/RSFC loadings of both models.

#### Multivariate ridge regression with structural lesion features

To understand the anatomical basis of the FC–deficit LC, we used a ridge regression machine learning algorithm to explore whether structural lesion features could predict FC or behavioural composite scores obtained from the first PLSC analysis. The structural lesion features included region-based SDC measures and region-based lesion damage. Features with zero values across all participants were removed from the analysis. All ridge regression models were trained and tested via leave-one-out cross-validation (LOOCV). In each loop, the regularisation coefficient alpha was optimized by identifying a value between *α* = 2^−10^ and 2^5^ that minimized leave-one-out prediction error over the training set. Next, optimal weights were calculated across the entire training set via gradient descent to minimize error in the ridge regression equation. These optimal weights were then applied to the SDC/lesion features of the left-out participants to predict behavioural or RSFC composite scores. A prediction was generated for all participants with this procedure. Model performance was evaluated by calculating Pearson's correlation coefficient between the measured and predicted composite scores.

#### Mediation analysis among the RSFC, SDC and behaviour composite scores

We used mediation analysis to explore the complex relationships among SDC, RSFC and behaviours. In the first mediation analysis, the RSFC composite scores from the FC–deficit PLSC model were defined as the mediator variable, the SDC composite scores from the SDC–deficit PLSC model were defined as the independent variable, and the behavioural composite scores from both PLSC models were defined individually as the dependent variable. In the second mediation analysis, the SDC composite scores were defined as the mediator variable, the FC composite scores were defined as the independent variable, and the behavioural composite scores were defined individually as the dependent variable. Theoretically, the relationship between the SDC and behaviour is mediated by large-scale functional connectivity disruptions. Therefore, we hypothesized that the mediation effect would be significant in the first analysis but not in the second.

We employed a non-parametric bootstrapping method to assess the significance of the mediation effect. After 10 000 bias-corrected bootstraps, we estimated the distribution of the indirect effect and calculated its 95% confidence interval (CI). A mediation effect was considered significant (*P* < 0.05) if the 95% CI did not contain zero. As mediators and dependent variables were assessed simultaneously, the mediation results should be interpreted as statistical associations rather than causal effects.

#### Control and reliability analysis

Several analyses were performed to ensure the robustness of our results. First, we constructed networks on the basis of an alternative parcellation developed by Yan *et al.*^45^ to demonstrate the robustness of the results independent of parcellation choices. FC was computed for 400 cortical regions in 7 large-scale networks. Second, we performed LOOCV of the PLSC analysis. In each fold, one patient was selected as the test set, while the remaining patients were selected as the training set. For the training set, PLSC was computed to obtain brain and behavioural saliences. We subsequently computed the brain and behavioural composite scores of the test patients by projecting the test data onto the saliences derived from the training data. Each patient served as a test patient in 1-fold of the analysis. We subsequently computed Pearson's correlations between the brain and behavioural composite scores of test patients across all folds and tested the statistical significance of these correlations via a permutation test (1000 times). Third, we also conducted internal validation by randomly splitting the dataset into two independent subsets (41 and 40 participants, respectively) and performing separate PLSC analyses on each subset. Fourth, to reduce feature dimensionality and ensure the robustness of our findings, we employed two distinct approaches. Principal component analysis (PCA) was used to reduce the dimensionality of input features prior to partial least squares (PLS) analysis. Sparse PLS (SPLS) embedded L1-norm regularisation into the PLS framework to enable automatic feature selection. Finally, we regressed out the confounding effects, including age, sex, ethnicity, lesion types and smoking status from both the brain and behaviour data before the PLSC analysis.

## Results

### One robust LC links RSFC and behaviour 2 weeks after stroke

**Figure 2:**
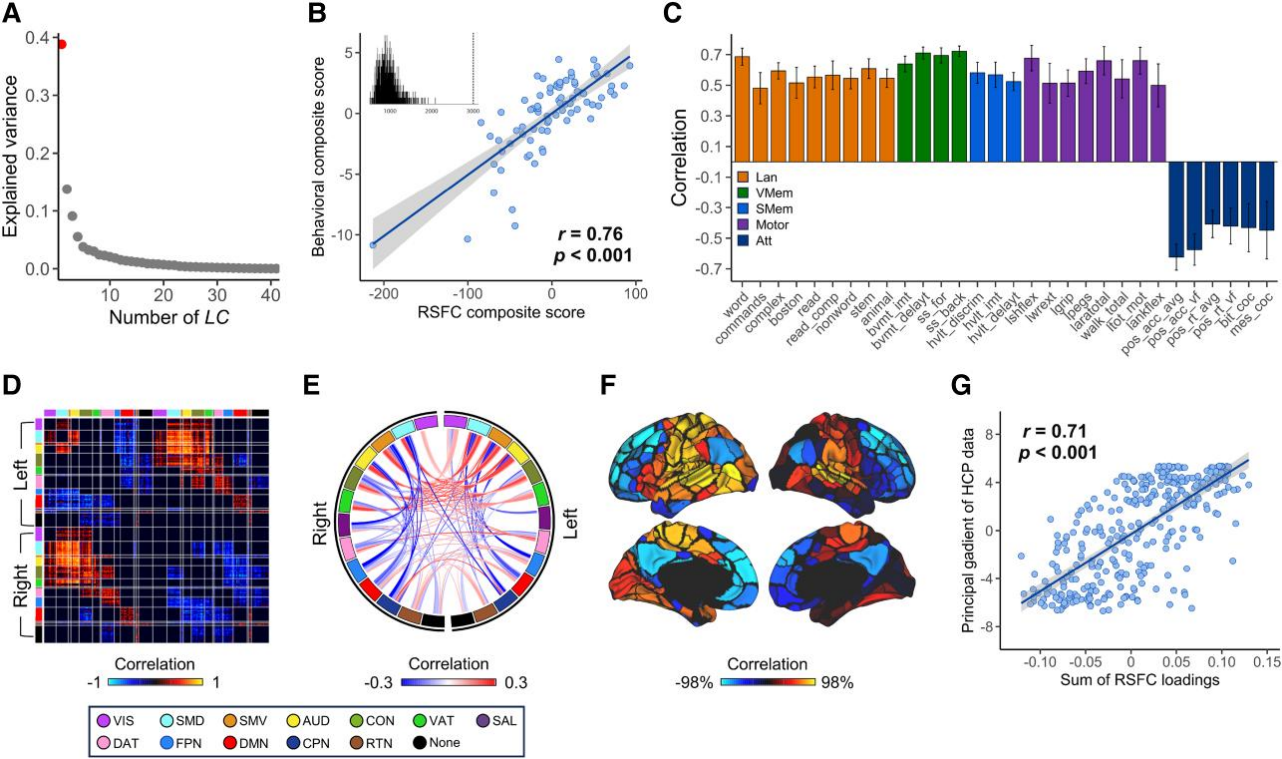
**One robust LC linking whole-brain RSFC with multiple neuropsychological scores 2 weeks after stroke.** (**A**) The amount of covariance explained by each LC. Each dot represents an LC, and the red dot represents the first LC, which is the only component that survived after permutation testing with FDR correction (*q* < 0.05). (**B**) Correlations between individual-specific RSFC and behavioural composite scores (*P* < 0.001) (*n* = 81). The inset shows the null distribution obtained by permutation testing. Note that the null distribution is not centred at zero. The dashed line indicates the actual singular value for LC1. (**C**) The 30 most significant correlations between behavioural measures and behavioural composite scores. The colours of the bars indicate different behavioural domains. The error bars indicate bootstrapped standard deviations with 1000 bootstrap estimations. (**D**) Significant correlations between RSFC data and RSFC composite scores; only within-network or between-network blocks with significant bootstrapped *Z* scores are shown (*q* < 0.05). Red (or blue) colour indicates that greater RSFC is positively (or negatively) associated with LC1. (**E**) Correlations between RSFC data and RSFC composite scores of participants, averaged within and between networks with significant bootstrapped *Z* scores. (**F**) Summed RSFC loadings for each cortical parcellation to represent the regional importance scores. (**G**) Correlation between the brain region map of summed RSFC loadings and the principal gradient of functional connectivity in healthy humans^69^ (*P* < 0.001). Each dot represents a cortical parcellation (*n* = 333). Att, attention; AUD, auditory; CON, cingulo-opercular network; CPN, cingulo-parietal network; DAT, dorsal attention; DMN, default mode network; FPN, frontoparietal network; Lan, language; RTN, retrosplenial temporal network; SAL, salience; SMD, somatomotor dorsal; SMem, spatial memory; SMV, somatomotor ventral; VAT, ventral attention; VIS, visual; VMem, verbal memory.

We conducted PLSC analysis on whole-brain RSFC data and 41 neuropsychological measures collected 2 weeks after stroke. Given that only the first latent component (LC1) survived permutation testing with false discovery rate (FDR) correction (*q* < 0.05), LC1 is the focus of the remainder of this article. LC1 accounted for 38.8% of the covariance between the RSFC and behavioural data, with a significant association (*r* = 0.76, *P* < 0.001) between the behavioural and RSFC composite scores (Fig. 2A and B).

Figure 2C shows the top correlations between the behavioural composite scores of LC1 and the 30 significant behavioural measures (thresholded behavioural loadings). A greater behavioural composite score was associated with greater general-domain behavioural performance (e.g. attention, verbal memory, spatial memory, language, motor). Unthresholded behavioural and RSFC loading patterns are shown in Supplementary Fig. 1. Notably, the accuracy of disengagement effect in the Posner orienting task showed a positive correlation, in contrast to the predominantly negative trend among other attention-domain measures. Figure 2D illustrates the significant correlations between the RSFC composite scores of LC1 and the RSFC among 333 cortical parcellations (thresholded RSFC loadings). The significant RSFC loadings were averaged within and between networks (Fig. 2E). A greater RSFC composite score was associated with increased interhemispheric RSFC within the sensorimotor (visual, auditory and somatomotor), cingulo–opercular, and attention networks. Conversely, a greater composite score was associated with decreased intrahemispheric connectivity between the default mode network or frontoparietal network and other networks. We further summed the RSFC loadings for each region in the Gordon atlas to represent the relative importance of each region in the significant RSFC pattern (Fig. 2F). The FC pattern of general-domain deficits followed the hierarchy gradient of cortical organisation (Fig. 2G). In other words, post-stroke behavioural performance was associated with divergent modulation of FC across cortical hierarchies between higher-order cognitive networks and lower-order sensorimotor regions.

### Replicability and generalizability across stroke stage

**Figure 3:**
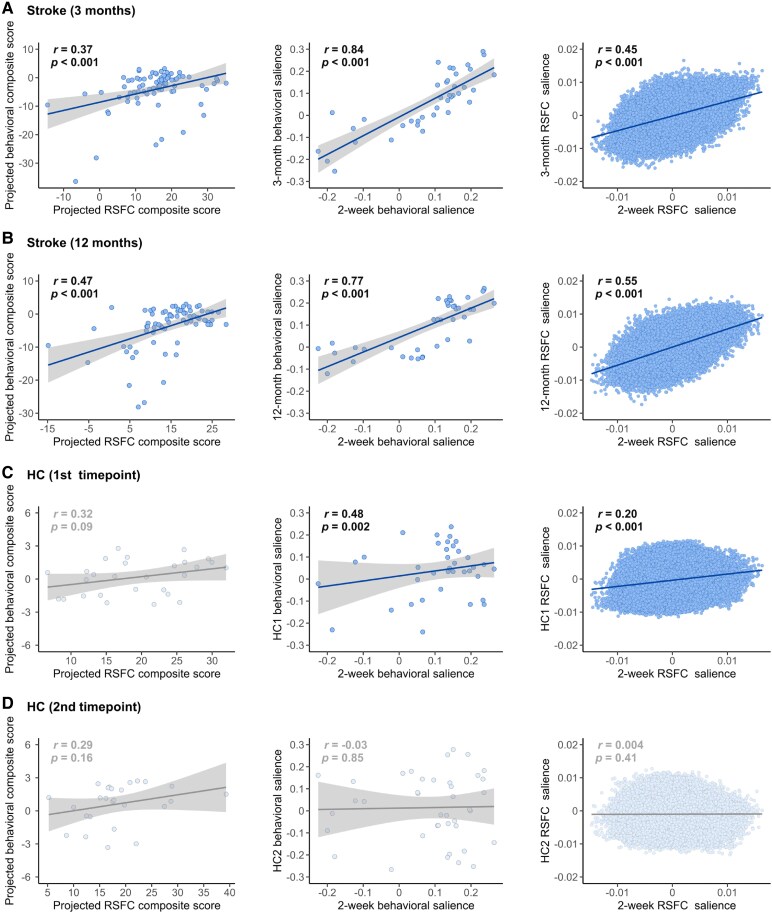
**The LC1 obtained from 2-week stroke data was verified with 3-month and 12-month stroke data, as well as HC data.** (**A**) Comparison between 2-week and 3-month stroke data. The left scatter plot shows the Pearson correlation between the behavioural and RSFC composite scores by projecting 3-month stroke data onto the salience parameters learned by PLSC on 2-week stroke data (*P* < 0.001). Each point represents a 3-month stroke patient (*n* = 78). The middle scatter plot shows the Pearson correlation between behavioural saliences in the 2-week and 3-month stroke data (*P* < 0.001). Each point represents a behavioural test. The right scatter plot shows the Pearson correlation between the RSFC saliences in the 2-week and 3-month stroke data (*P* < 0.001). Each point represents an edge between two cortical regions. (**B**) Comparison of 2-week and 12-month stroke data. The left scatter plot shows the correlation between the behavioural and RSFC composite scores by projecting 12-month stroke data onto the salience parameters from 2-week stroke data (*P* < 0.001). Each point represents a 12-month stroke patient (*n* = 74). The middle scatter plot shows the correlation between behavioural saliences in the 2-week and 12-month stroke data (*P* < 0.001). The right scatter plot shows the correlation between the RSFC saliences in the 2-week and 12-month stroke data (*P* < 0.001). (**C**) Comparison between 2-week stroke data and control data at the first visit (HC1). The left scatter plot shows the correlation between the behavioural and RSFC composite scores by projecting HC1 data onto the salience parameters from 2-week stroke data (*P* > 0.05). Each point represents a HC1 participant (*n* = 28). The middle scatter plot shows the correlation between behavioural saliences in the 2-week stroke data and HC1 data (*P* > 0.05). The right scatter plot shows the correlation between RSFC saliences in the 2-week stroke data and HC1 data (*P* < 0.001). (**D**) Comparison between 2-week stroke data and control data at the second visit (HC2). The left scatter plot shows the correlation between the behavioural and RSFC composite scores by projecting HC2 data onto the salience parameters from 2-week stroke data (*P* > 0.05). Each point represents a HC2 patient (*n* = 26). The middle scatter plot shows the correlation between behavioural saliences in the 2-week stroke data and HC2 data (*P* > 0.05). The right scatter plot shows the correlation between the RSFC saliences in the 2-week stroke data and HC2 data (*P* > 0.05).

The LC1 identified from 2-week stroke data was further validated with stroke data at 3-month and 12-month time points. We applied PLSC to whole-brain RSFC and 41 neuropsychological assessments at 3-month and 12-month points. The PLSC analysis consistently revealed that LC1 was significant at both time points (3-month: *r* = 0.82, permuted *P* = 0.018; 12-month: *r* = 0.79, permuted *P* = 0.023), explaining 23.0%, 23.2% of covariance between the brain and behavioural data, respectively (Supplementary Fig. 2A and B). Importantly, the LC1 obtained from 3-month and 12-month stroke data largely replicated the LC1 identified from 2-week stroke data. This is evidenced by the high correlation between behavioural saliences in the 2-week and 3-month stroke data (*r* = 0.84, *P* < 0.001), between behavioural saliences in the 2-week and 12-month stroke data (*r* = 0.77, *P* < 0.001), between RSFC saliences in the 2-week and 3-month stroke data (*r* = 0.45, *P* < 0.001), between RSFC saliences in the 2-week and 12-month stroke data (*r* = 0.55, *P* < 0.001) (Fig. 3A and B). The correlation results of loadings were consistent with the results of saliences (Supplementary Fig. 3).

We further performed a cross-stage validation analysis by projecting stroke data at 3 months and 12 months onto the salience parameters learned by PLS in stroke data at 2 weeks, and then calculated the correlation between the behavioural and RSFC composite scores in projected data. We found that the obtained LC1 from stroke data at 2weeks successfully generalized to 3-month and 12-month stroke data, as evidenced by a significant correlation between the obtained behavioural and RSFC composite score (3-month: *r* = 0.37, *P* < 0.001; 12-month: *r* = 0.47, *P* < 0.001) (Fig. 3A and B).

Moreover, we applied PLSC to whole-brain RSFC and raw behavioural measures of healthy controls. The first LC was significant for the control group for each visit (visit 1: *r* = 0.880, permuted *P* < 0.001; visit 2: *r* = 0.886, permuted *P* = 0.025) (Supplementary Fig. 2C and D). But correlations between behavioural and RSFC saliences of the 2-week stroke group and control group were low, indicating the LC1 obtained from the control data is distinct from that of the stroke data (Fig. 3C and D). Moreover, when we projected HC data onto the salience parameters learned by PLS in 2-week stroke data, the correlations between the behavioural and RSFC composite score were non-significant (Fig. 3C and D). These results suggest the obtained LC1 can only be replicated and generalized using stroke data rather than control data.

### Structural disconnection better explains FC–deficit LC1 scores than cortical damage

**Figure 4:**
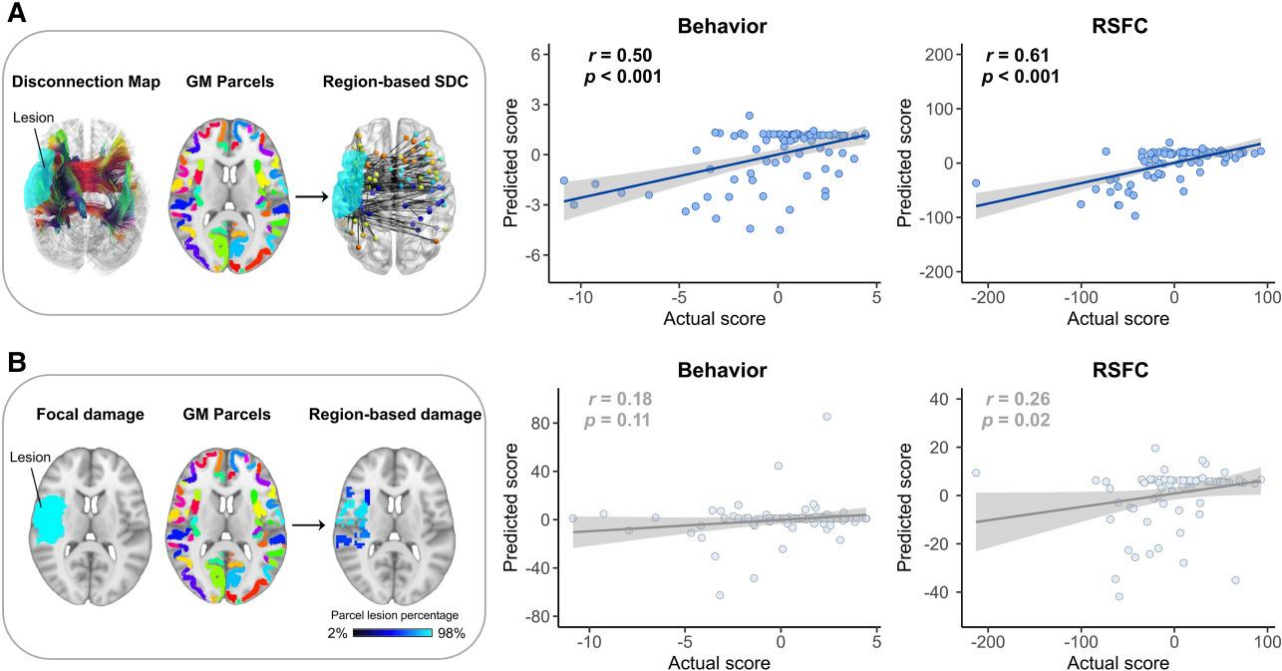
**Prediction performance of structural lesion features.** (**A**) Left: A disconnection streamline map, a brain parcellation map and a structural connectome atlas are used to create a region-based SDC matrix, where each edge represents the proportion of streamlines connecting two regions that are disconnected due to the lesion. Right: Correlations between the predicted and real behaviour/RSFC composite scores when region-based SDC is a predictor (*P* < 0.001). (**B**) Left: Lesion topography and brain parcellation are used to compute a region-based damage vector, where each value indicates the percentage of voxels within each grey matter parcel that were damaged by the lesion. Right: Correlations between the predicted and real behaviour/RSFC composite scores when lesion damage is a predictor (*P* > 0.05). Each point represents a 2-month stroke patient (*n* = 81).

To explore the anatomical basis of LC1, we utilized a ridge regression model to predict the FC–deficit composite scores using region-based lesion damage and SDC. When we predicted the composite scores using SDC, the prediction performance was significant, as evidenced by the correlation between the predicted and test composite behavioural scores (*r* = 0.50, *P* < 0.001, *R*^2^ = 0.25, MAE = 2.01) and between the predicted and test composite RSFC scores (*r* = 0.61, *P* < 0.001; *R*^2^ = 0.37, MAE = 28.11) after LOOCV (Fig. 4A). However, when the composite scores were predicted using focal lesion damage, the prediction performance was not significant, as evidenced by the correlation between the predicted and test composite behaviour scores (*r* = 0.18, *P* = 0.11; *R*^2^ = −0.59, MAE = 4.67) and between the predicted and test composite RSFC scores (*r* = 0.26, *P* = 0.02; *R*^2^ = 0.05, MAE = 34.09) after LOOCV (Fig. 4B). SDC features outperformed lesion damage features in explaining the core mode of covariation between FC and deficits, indicating that SDC of white matter pathways is the primary anatomical factor underlying the association between FC and multiple deficits. To characterize the contributions of SDC, the top 20% of the strongest weights were projected back onto the brain (Supplementary Fig. 4), showing that FC–deficit scores were negatively correlated with interhemispheric SDC across most networks, such as the sensorimotor, cingulo–opercular, attention and default networks.

### Mediating role of FC in the relationship between SDC and behaviour

**Figure 5:**
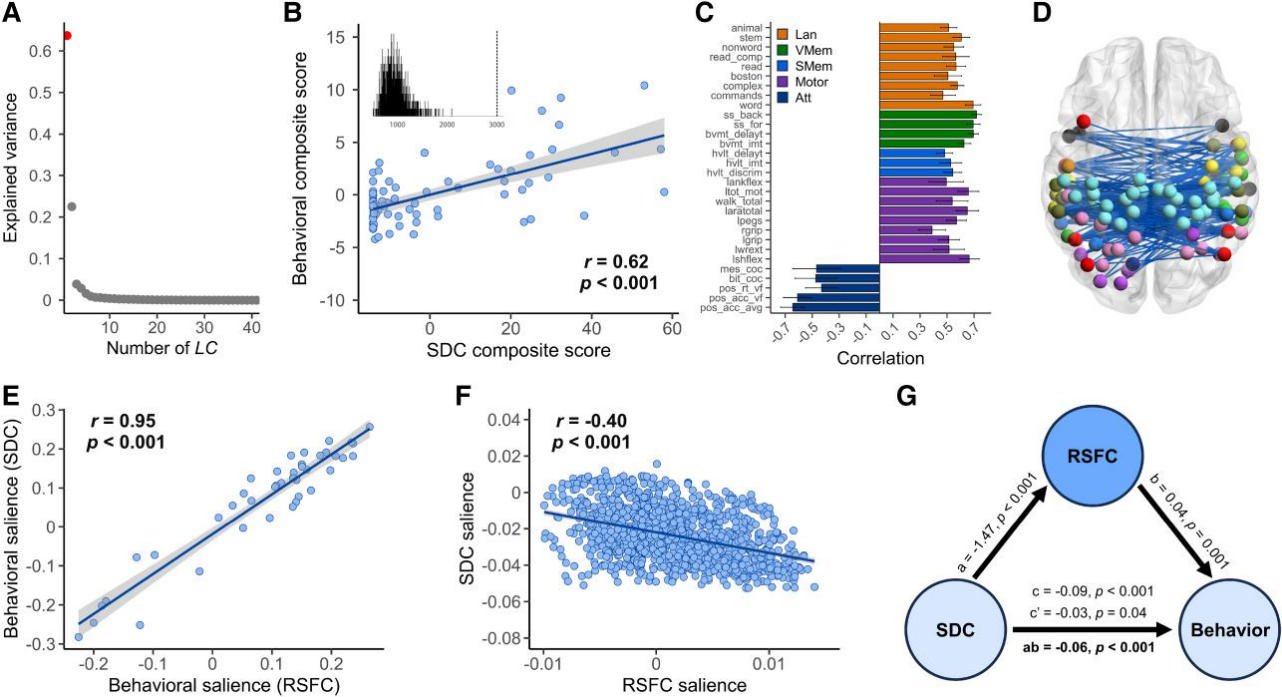
**One robust component linking structural disconnection with raw behavioural assessments and its correlation with the LC1 obtained from the FC–deficit PLSC model.** (**A**) The amount of covariance explained by each LC. Each dot represents an LC, and the red dot represents the LC that survived after permutation testing with FDR correction (*q* < 0.05). (**B**) Correlations between individual-specific SDC and behavioural composite scores (*n* = 81). The inset shows the null distribution obtained by permutation testing. Note that the null distribution is not centred at zero. The dashed line indicates the actual singular value obtained for LC1. (**C**) The 30 strongest correlations between behavioural measures and behavioural composite scores. The error bars indicate bootstrapped standard deviations with 1000 bootstrap estimations (*n* = 1000). (**D**) The top 20% of correlations between SDC data and SDC composite scores. The blue colour indicates that SDC is negatively associated with LC1. (**E**) The correlation between behavioural saliences obtained from the SDC–deficit PLSC model and those obtained from the FC–deficit PLSC model (*P* < 0.001). (**F**) The correlation between SDC saliences from the SDC–deficit PLSC model and RSFC saliences from the FC–deficit PLSC model (*P* < 0.001). (**G**) Mediation analysis for SDC composite scores (independent variable), RSFC composite scores (mediating variable) and behaviour composite scores (dependent variable). The total effect (c = −0.09, *P* < 0.001) and indirect effect (ab = −0.06, *P* < 0.001) are very significant, and the direct effect (c′ = −0.03, *P* = 0.04) is significant.

We conducted PLSC analysis on region-based SDC and 41 neuropsychological measures at the 2-week post-stroke. The PLSC analysis revealed that LC1 was significant (*q* < 0.05); LC1 accounted for 63.7% of the SDC–deficit covariance, with a significant association (*r* = 0.62, *P* < 0.001) between the behavioural and SDC composite scores (Fig. 5A and B). Figure 5C shows the top correlations between the behavioural composite scores and behavioural data of LC1. Figure 5D shows the top 20% of SDC loadings, primarily consisting of interhemispheric SDCs within the sensorimotor, cingulo-opercular, attention and default network. Notably, the behavioural patterns driven by SDC are highly correlated with previous behavioural patterns driven by FC (Fig. 5E), indicating that the LC1 also reflects general cognitive performance. The SDC patterns derived from the SDC–behaviour data were highly correlated with the FC patterns derived from the FC–behaviour data (Fig. 5F), supporting that SDC may underlie core FC disruptions associated with stroke.

We performed a mediation analysis using RSFC composite scores as the mediating variable, SDC composite scores as the independent variable, and behaviour composite scores as the dependent variable. The results showed that FC scores partially mediated the relationship between the SDC and behaviour scores [ab = −0.06, 95% confidence interval (CI) (−0.09, −0.04)] (Fig. 5G). The total effect (c) was −0.09, and the proportion of mediation (ab/c) was 66.7%. However, no mediating effect was observed when the mediating variable was the SDC composite score, the independent variable was the RSFC composite score, and the dependent variable was the behaviour composite score [ab = −0.01, 95% CI (0, 0.02)]. The total effect (c) was −0.04, and the proportion of mediation (ab/c) was 25%. These results offer compelling evidence that large-scale disruption of FC mediates the relationship between SDC and multidomain behavioural deficits from a data-driven perspective.

### Control and reliability analyses

We performed several additional analyses to ensure the robustness of our results. First, in LOOCV, the PLSC components estimated from training participants were successfully generalized to the remaining participants (Supplementary Fig. 5). Second, the first LC identified in the full dataset was stable across different subsamples. Third, the results were largely unchanged when the RSFC matrix and region-based structural features were based on an alternative parcellation developed by Yan *et al.*^45^ (Supplementary Figs. 6–8). Fourth, to ensure that the results were not affected by the interpolation processing method for missing behavioural values, we conducted all analyses after excluding participants with incomplete behavioural data. All the results were unchanged. Fifth, the first LC remained stable regardless of whether PCA or SPLS was used for dimensionality reduction. Finally, the results were replicated after we regressed out the confounding effects from both the RSFC and behaviour data.

## Discussion

Although existing studies have explored the relationship between whole-brain RSFC and neurological deficits after stroke, the degree to which RSFC is associated with deficits as a multivariate dimension remains largely unexplored. Employing a multivariate data-driven approach, we discovered covariance patterns of RSFC and neurological deficits that were represented by the LC1. This component shows that the severity of behavioural deficits across multiple domains is associated with decreased interhemispheric connectivity and increased intrahemispheric connectivity. Notably, the LC was maintained over the course of recovery from stroke. Furthermore, this component was better explained by damage to underlying white matter tracts than by focal lesions. Then we examined the relationships among SDC, RSFC and deficits and found that RSFC might mediate the relationship between SDC and multidomain deficits.

Previous studies applied dimensionality reduction techniques to an extensive battery of post-stroke behavioural measures and revealed that three robust clusters accounted for most of the variance across behavioural domains.^23^ These behavioural clusters are considered possible representations of the behavioural output of a common, abnormal functional network state; however, this hypothesis lacks substantial empirical support.^12^ In the present study, we identified a comprehensive behavioural cluster by incorporating whole-brain RSFC. This cluster involved 30 neurological measures across five behavioural domains (including attention, language, spatial memory, verbal memory, and motor), reflecting general-domain cognitive performance after stroke. Notably, the accuracy of disengagement effect in the Posner orienting task showed a positive correlation with the FC–behaviour dimension, in contrast to the trend of other attention-domain scores (Supplementary Fig. 1). This opposite direction may reflect the distinct neurocognitive basis of disengagement, which is strongly right-lateralized and particularly vulnerable to right-hemisphere damage.^46,47^ Taken together, these results provide evidence supporting the hypothesis that highly correlated deficits resulted from abnormal patterns of synchronisation in functional networks.

The abnormal FC pattern was characterized by reduced interhemispheric and increased intrahemispheric connectivity. Such a pattern is consistent with previous research, which has demonstrated its strong association with specific behavioural scores, including motor, language, attention and memory performance.^20,22^ Researchers have suggested that these alterations in FC may be related to a significant reduction in the modularity of the whole-brain network compared with healthy adults.^40,48^ From a more theoretical perspective, these FC abnormalities following a stroke may reflect a decrease in the entropy of the brain and the variability of neural states, which could correspond to a decrease in the number of possible behaviours that can be generated.^49-51^ Furthermore, we discovered that the abnormal FC pattern aligns with the brain's sensorimotor-association (S-A) axis. The more severe the behavioural deficits are, the weaker the connectivity in lower-level sensory networks and the stronger the connectivity in higher-level cognitive networks. A series of studies have consistently revealed that FC patterns linked to overall behavioural scores (such as the general psychopathology factor) follow a hierarchical gradient from lower-order to higher-order regions.^52-54^ Lower-order and higher-order regions, which exhibit opposite modulations, are known to be structurally and functionally distinct. These two types of brain regions are structurally different in terms of cortical myelination, cortical layering, cytoarchitecture, cell types, and synaptic physiology, and they serve either simple sensorimotor functions or complex abstract cognitive functions.^55,56^ Together with previous findings, our results demonstrate that lower-order and higher-order regions exhibit opposite effects on general behavioural performance after stroke.

The covariance patterns between deficits and FC in the LC1 were maintained up to 12 months after stroke but did not exist in the HC data, which demonstrated the robustness and specificity of the identified component. One possible explanation is that the recovery of multiple functions depends on common recovery mechanisms after stroke. As previous behavioural studies reported, the time course and magnitude of recovery were similar across behavioural domains and the covariance structure of behavioural deficits remained stable during recovery from stroke.^24,57-60^ These shared mechanisms involve fundamental plasticity processes including the restoration of global functional connectivity patterns and the rebalancing of network-level excitation-inhibition dynamics.^21,61^ While our findings support domain-general mechanisms, we acknowledge the coexistence of domain-specific adaptations. For example, motor recovery frequently involves topographic reorganisation of perilesional cortex; language recovery may engage distinct patterns of contralateral homologue recruitment; attention deficits show particular dependence on the resynchronisation of specific attention networks.^62^ This evidence suggests that post-stroke recovery involves both local network plasticity and overarching biological repair processes, which may operate in parallel or interact in complex ways depending on the functional domain.

We observed a slightly higher correlation between 2-week and 12-month data compared with that between 2-week and 3-month data. While a stronger correlation at the shorter interval might be expected, this seemingly counterintuitive pattern likely reflects the dynamic and non-linear nature of post-stroke recovery. The 3-month stage typically represents a transitional period marked by rapid and heterogeneous changes in both behaviour and functional connectivity, which may weaken alignment with early brain–behaviour associations. In contrast, recovery tends to stabilize by 12 months, and individual patterns become more consistent, facilitating stronger correspondence with the early LC.

Furthermore, SDC features consistently outperformed focal damage features in predicting FC–deficit PLSC scores, highlighting SDC as a critical structural basis for FC–deficit associations. Previous studies have proposed that damage to hub regions connecting multiple functional networks leads to widespread FC disruptions and extensive behaviour impairments.^35,48^ However, the severe effect of damage to certain cortical hubs might be better explained by damage to the underlying white matter tracts, as SDC contain more complex damage information than focal lesions.^12,23,34^ A recent study demonstrated that indirect SDC of grey matter regions better explained widespread FC abnormalities than focal lesions did.^33^ In a healthy human brain, the efficacy of cognitive processes relies on communication between multiple areas and damage to the major white matter tracts facilitating this communication is likely to cause multiple deficits.^13^ Notably, white matter disconnection has a long-lasting detrimental effect and constrains functional and behavioural outcomes. This may partially explain why the deficit–FC LC identified at 2 weeks persists across recovery.

Many studies on white matter damage have associated specific behavioural deficits with SDC.^34,63-65^ Our study identified a significant LC linking SDC and an extensive battery of deficits across multiple domains. The behavioural pattern of this component, which is highly similar to the pattern obtained from FC–deficit PLSC analysis, reflects general-domain cognitive performance. This component consists of interhemispheric SDCs, which have been demonstrated to be a key factor in the imbalance of large-scale functional networks. For example, studies of non-human primates have suggested that transection of the corpus callosum results in a decrease in between-hemisphere FC and an increase in within-hemisphere FC.^66,67^ A human study also revealed that the severity of interhemispheric SDC was correlated with both type of abnormal FC patterns.^33^ Taken together, interhemispheric SDC, multi-domain deficits and two abnormal patterns of FC are closely linked. However, prior studies have not thoroughly examined the complete relationships among SDC, FC and multidomain deficits within a unified analytical framework. The present study conducted a mediation analysis using the PLSC composite score of the three variables and revealed that FC statistically mediated the association between SDC and behavioural deficits. It provided a deeper understanding of interrelationships among the three variables.

In this study, we discovered and replicated multivariate patterns of intrinsic FC that are highly correlated with a dimension of neuropsychological deficit in a longitudinal cohort of stroke patients. The dimension was composed of a common post-stroke feature of FC and a unique cluster of deficits involving multiple domains. This multivariate framework moves beyond pairwise associations and reveals a large-scale network-level mechanism that may underlie inter-individual differences in recovery trajectories following stroke. Furthermore, we found that SDC is closely associated with this FC–deficit dimension, which provides empirical evidence for the systematic interaction between SDC, FC, and multi-domain behavioural deficits.^12^ Rather than replicating prior findings, our study offers novel insights by demonstrating that FC statistically mediates the relationship between SDC and multidomain deficits. Clinically, these findings have potential applications in prognosis and targeted interventions. Specifically, the FC–deficit dimension could serve as a neurophysiological biomarker to monitor recovery and inform personalized rehabilitation. Functional connectivity assessments could help tailor adaptive therapy protocols, focusing on task-specific training that targets network dysfunction rather than traditional motor or cognitive exercises. Moreover, our findings support the use of multisite neuromodulation strategies (e.g. TMS, tDCS) that target network-level dysfunctions rather than isolated regions, potentially improving treatment efficacy.

There are several limitations to this study. First, the relatively small sample sizes in both the stroke and control groups may increase the risk of overfitting, particularly in multivariate analyses. To reduce the dimensionality of input features, we used PCA and SPLS to validate the stability of the PLS results. While these approaches reinforce the internal robustness of our findings, the generalizability of PLS outcomes remains limited in the absence of external validation. Moreover, the predictive framework based on LOOCV is inherently constrained by the small sample size and available data, which restricts the generalizability of the results to independent datasets. Future studies involving larger, independent stroke cohorts will be essential to further assess the reproducibility and external validity of these multivariant models. Second, we focused on unilateral stroke patients and excluded those with bilateral lesions. This decision aimed to reduce sample heterogeneity and ensure the stability and interpretability of multivariate analyses. Bilateral strokes often involve more complex and widespread damage, which may introduce additional sources of variance that are difficult to model in relatively small samples.^68^ However, we acknowledge that this choice may limit the generalizability of our findings to the broader stroke population. Future studies with larger and more diverse samples will be necessary to capture the full spectrum of network disruptions, including those associated with bilateral lesions. Finally, SDC measures were defined by intersecting patient lesions with a structural connectome atlas, which is assumed to approximate individual structural connectomes. These measures may not account for inter-individual variability in undamaged fibre pathways. While direct patient SDC measures would be ideal, existing studies suggest that indirect SDC data provide valuable information about structural connectivity after brain damage.^33,65^

## Supplementary Material

fcaf276_Supplementary_Data

## Data Availability

The full set of neuroimaging and behavioural data are available at http://cnda.wustl.edu/app/template/Login.vm. Data sharing is not applicable to this article as no new data were created in this study. Specific data and analysis scripts can be found at https://github.com/PsyWuke/Latent_dimension_linking_FC_with_post-stroke_deficits2025.git.
