# Correction to: Latent dimension linking functional connectivity with post-stroke deficits across multiple domains

**DOI:** 10.1093/braincomms/fcaf411

**Published:** 2025-10-27

Ke Wu, Yaya Jiang, Junhao Luo, Yijun Chen, Shaoling Peng, Xiangyu Kong, Gaolang Gong, Latent dimension linking functional connectivity with post-stroke deficits across multiple domains, *Brain Communications*, Volume 7, Issue 4, 2025, fcaf276, https://doi.org/10.1093/braincomms/fcaf276

A typographic error has been corrected in the graphical abstract of this article.

