# Supplementary material for: Latent dimension linking functional connectivity with post-stroke deficits across multiple domains
Source: Brain Commun. 2025 Jul 22;7(4):fcaf276. doi: 10.1093/braincomms/fcaf276 (PMC12308281; doi:10.1093/braincomms/fcaf276)
Supplement: fcaf276_Supplementary_Data [file fcaf276_supplementary_data.pdf]

Supplementary Materials for

Latent dimension linking functional connectivity with post-stroke deficits across multiple domains

This file includes:

Supplementary Fig. 1 to Supplementary Fig. 8
Supplementary Table 1

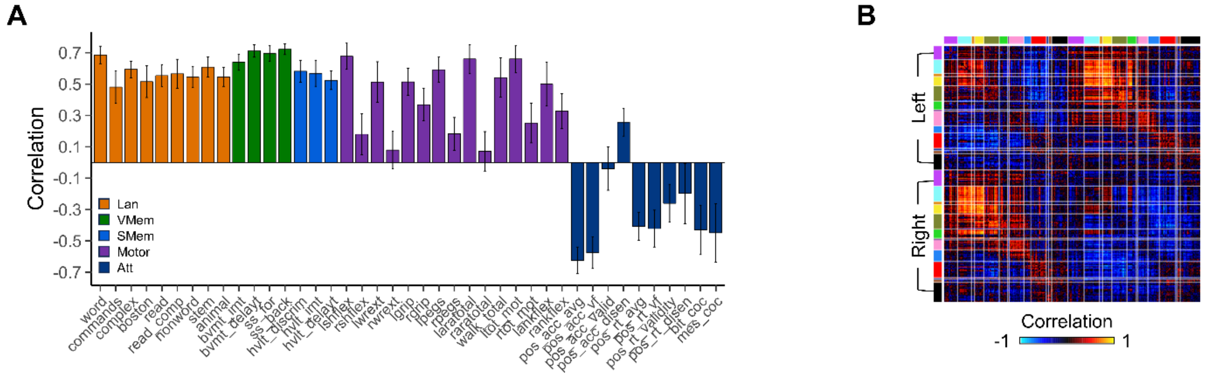

Supplementary Fig. 1. Unthresholded loadings. (**A**) All correlations between LC1 behavioral measures and behavioral composite scores. The colors of the bars indicate different behavioral domains. The error bars indicate bootstrapped standard deviations with 1000 bootstrap estimations. (**B**) All correlations between the LC1 RSFC data and RSFC composite scores. Red (or blue) color indicates that greater RSFC is positively (or negatively) associated with LC1.

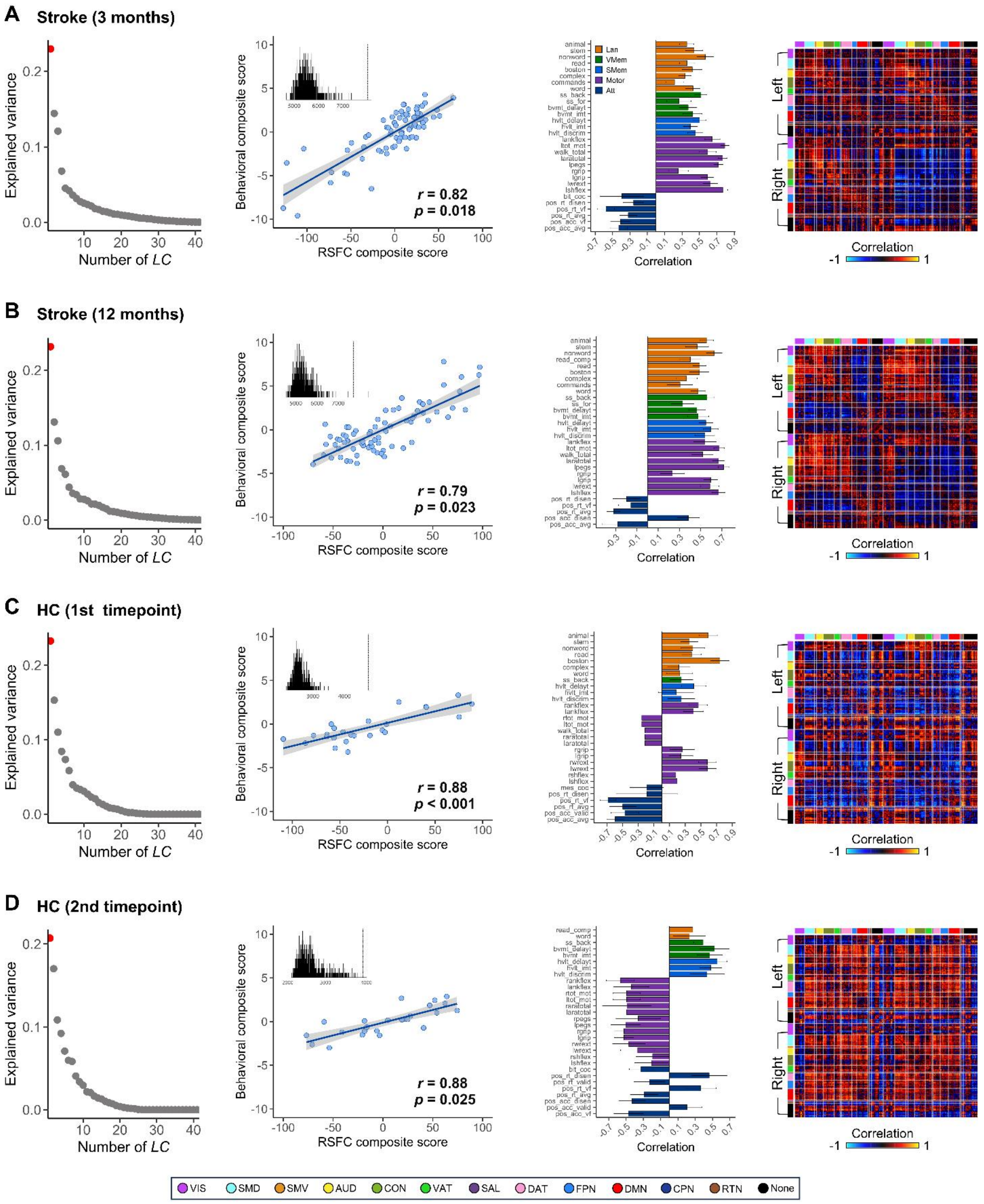

Supplementary Fig. 2. Robust LCs linking RSFC and raw neuropsychological scores for 3-month and 12-month stroke patient and healthy control data. (**A**) One robust LC obtained from 3-month stroke patients (n = 78). The first panel shows the amount of covariance explained by each latent component. The red dot represents the LC that survived after permutation testing. The second panel shows correlations between individual-specific RSFC and behavioral composite scores ($p < 0.05$). The inset shows the null distribution obtained by permutation testing. The third panel shows the 30 strongest correlations between the LC1 behavioral measures and behavioral composite scores. The error bars indicate bootstrapped standard deviations with 1000 bootstrap estimations. The fourth panel shows unthresholded correlations between participants' RSFC data and RSFC composite scores. Red (or blue) color indicates that greater RSFC is positively (or negatively) associated with LC1. (**B**) One robust LC obtained from 12-month stroke patients (n = 74). (**C**) One

robust LC obtained from healthy controls at the first visit (n = 28). (**D**) One robust LC obtained from healthy controls at the second visit (n = 26).

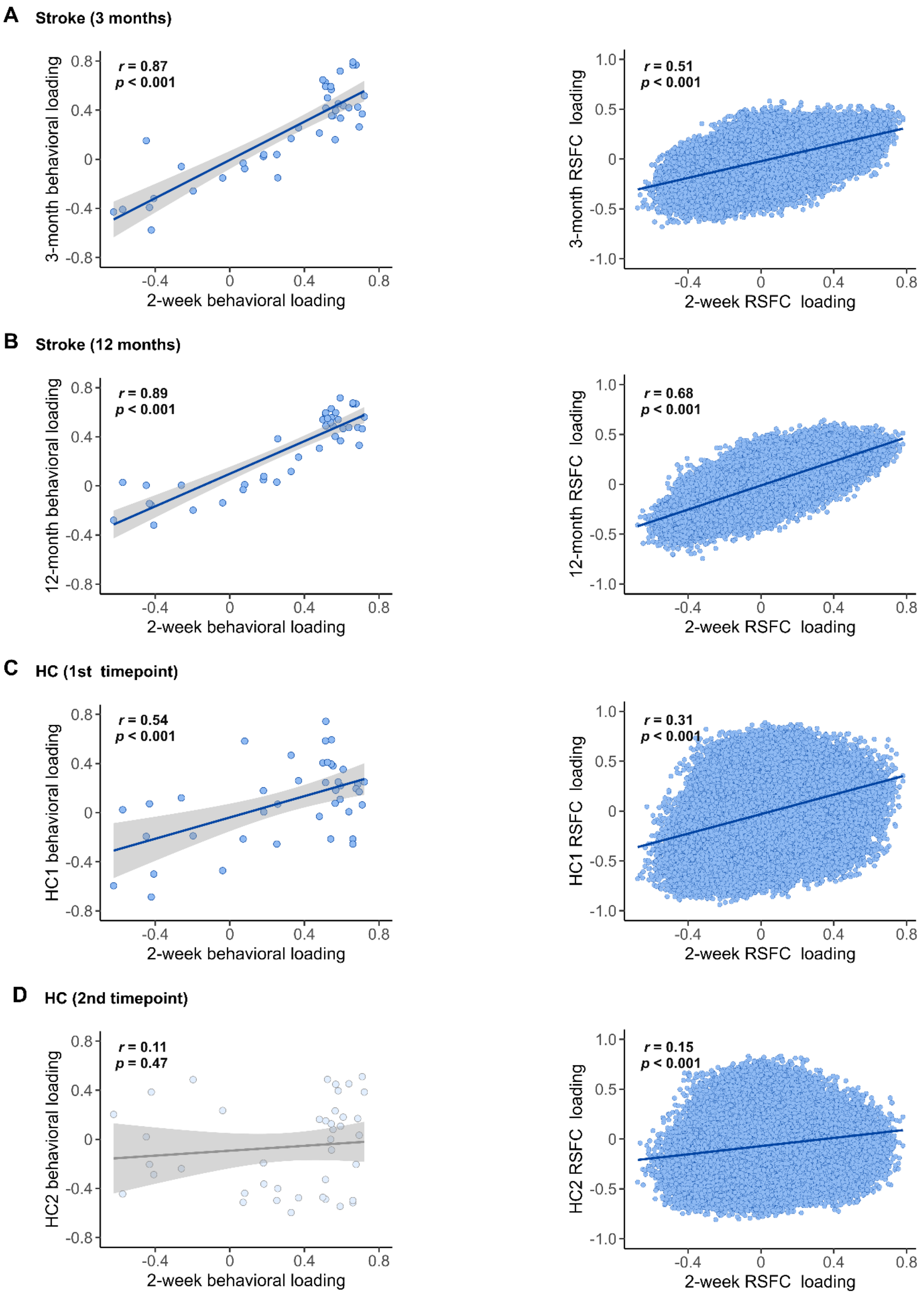

Supplementary Fig. 3. Replication of the LC1 loadings across 2-week stroke group and other groups. (A) The left scatter plot shows the Pearson correlation between behavioral loadings in the 2-week stroke group and 3-month stroke group ($r = 0.87$, $p < 0.001$). Each point represents a behavioral test (n = 41). The right scatter plot shows the Pearson correlation between the RSFC loadings in the 2-week stroke group and 3-month stroke

group ($r = 0.51$, $p < 0.001$). Each point represents an edge between two cortical regions (n = 55278). (**B**) The correlation between behavioral/RSFC loadings in the 2-week stroke group and 12-month stroke group ($r = 0.89$, $p < 0.001$; $r = 0.68$, $p < 0.001$). (**C**) The correlation between behavioral/RSFC loadings in the 2-week stroke group and HC1 group ($r = 0.54$, $p < 0.001$; $r = 0.31$, $p < 0.001$). (**D**) The correlation between behavioral/RSFC loadings in the 2-week stroke group and HC2 group ($r = 0.11$, $p > 0.05$; $r = 0.15$, $p < 0.001$).

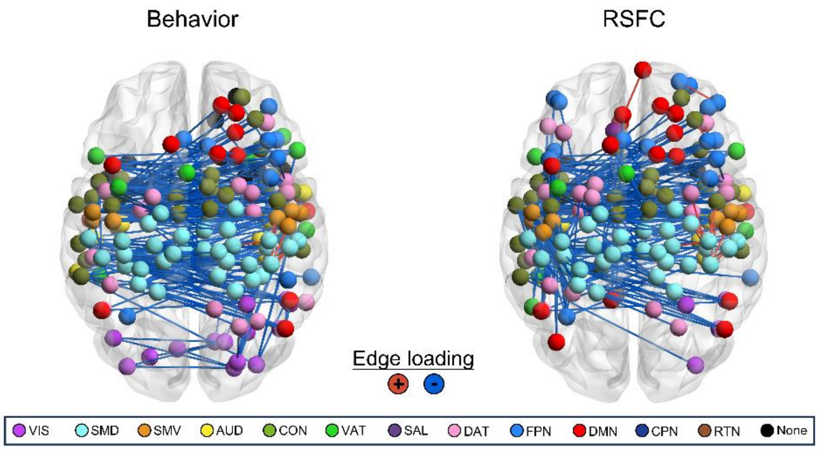

Supplementary Fig. 4. Predictive structural disconnections for the LC1 composite scores. Left: Top 20% of connections from the prediction model for the behavioral composite scores. Right: Top 20% of connections from the prediction model for the RSFC composite scores. A red edge indicates that severe structural disconnection yielded a higher composite score, and a blue edge indicates that severe structural disconnection yielded a lower composite score.

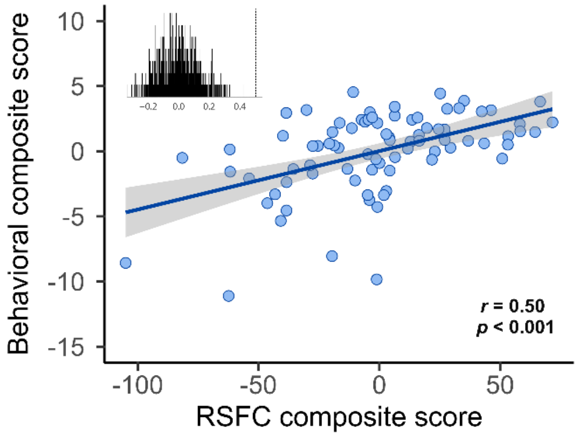

Supplementary Fig. 5. Leave-one-out cross-validation of the PLSC analysis. Pearson's correlations between the LC1 RSFC and behavioral composite scores of test patients across all folds (n = 81). The statistical significance of the correlations was tested via a permutation test (1000 times) ($p < 0.001$).

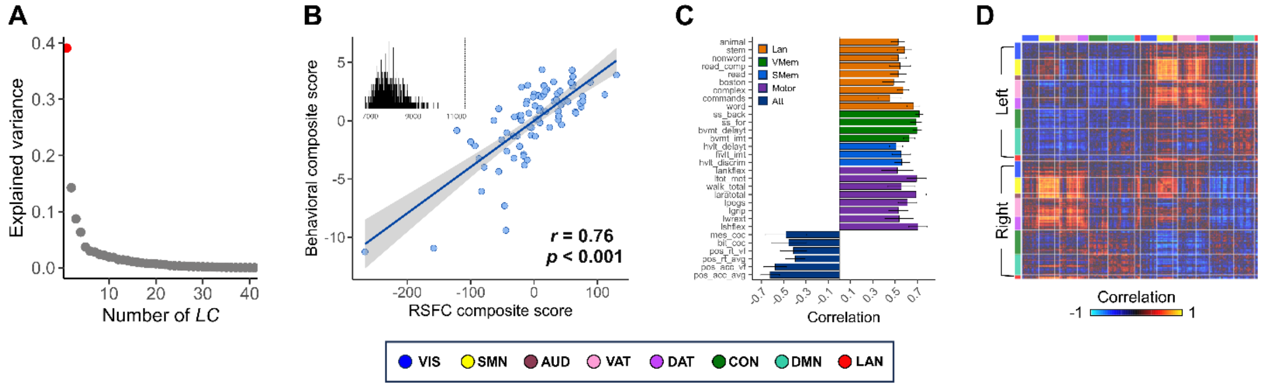

Supplementary Fig. 6. One robust LC linking the RSFC matrix constructed by alternative parcellation with multiple neuropsychological scores two weeks after stroke. (**A**) The amount of covariance explained by each LC. The red dot represents the LC that survived after permutation testing with FDR correction ($q < 0.05$). (**B**) Correlations between individual-specific RSFC and behavioral composite scores ($p < 0.001$) (n = 81). (**C**) The top 30 correlations between participants' behavioral measures and behavioral composite scores. (**D**) Unthresholded correlations between participants' RSFC data and RSFC composite scores. Red (or blue) color indicates that greater RSFC is positively (or negatively) associated with LC1.

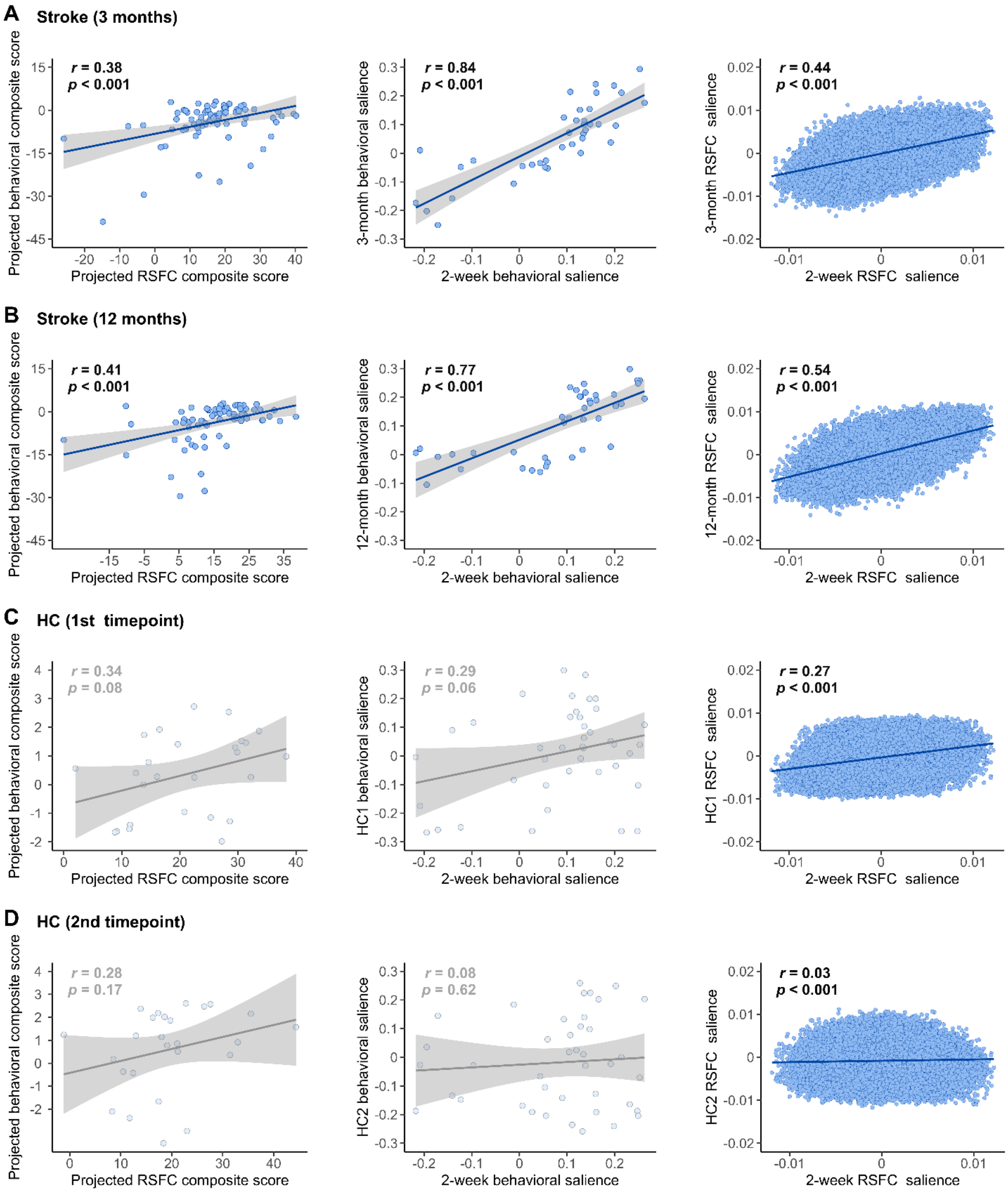

Supplementary Fig. 7. Generalization and replication of the LC1 obtained from two-week stroke data when the RSFC matrix was constructed by an alternative parcellation method. (A) Comparison between two-week and three-month stroke data. The left scatter plot shows the Pearson correlation between the behavioral and RSFC composite scores by projecting three-month stroke data onto the salience parameters learned by PLSC on two-week stroke data ($p < 0.001$). Each point represents a three-month stroke patient (n = 78). The middle scatter plot shows the Pearson correlation between behavioral saliences in the two-week and three-month stroke data ($p < 0.001$). Each point represents a behavioral test. The right scatter plot shows the Pearson correlation between the RSFC saliences in the two-week and three-month stroke data ($p < 0.001$). Each point represents an edge between two cortical regions. (B) Comparison of two-week and twelve-month stroke data. The left scatter plot shows the correlation between the behavioral and RSFC composite scores by projecting twelve-month stroke data onto the salience parameters from two-week stroke data ($p < 0.001$). Each

point represents a twelve-month stroke patient (n = 74). The middle scatter plot shows the correlation between behavioral saliences in the two-week and twelve-month stroke data ($p < 0.001$). The right scatter plot shows the correlation between the RSFC saliences in the two-week and twelve-month stroke data ($p < 0.001$). (C) Comparison between two-week stroke data and control data at the first visit (HC1). The left scatter plot shows the correlation between the behavioral and RSFC composite scores by projecting HC1 data onto the salience parameters from two-week stroke data ($p > 0.05$). Each point represents a HC1 patient (n = 28). The middle scatter plot shows the correlation between behavioral saliences in the two-week stroke data and HC1 data ($p > 0.05$). The right scatter plot shows the correlation between RSFC saliences in the two-week stroke data and HC1 data ($p < 0.001$). (D) Comparison between two-week stroke data and control data at the second visit (HC2). The left scatter plot shows the correlation between the behavioral and RSFC composite scores by projecting HC2 data onto the salience parameters from two-week stroke data ($p > 0.05$). Each point represents a HC2 patient (n = 26). The middle scatter plot shows the correlation between behavioral saliences in the two-week stroke data and HC2 data ($p > 0.05$). The right scatter plot shows the correlation between the RSFC saliences in the two-week stroke data and HC2 data ($p < 0.001$).

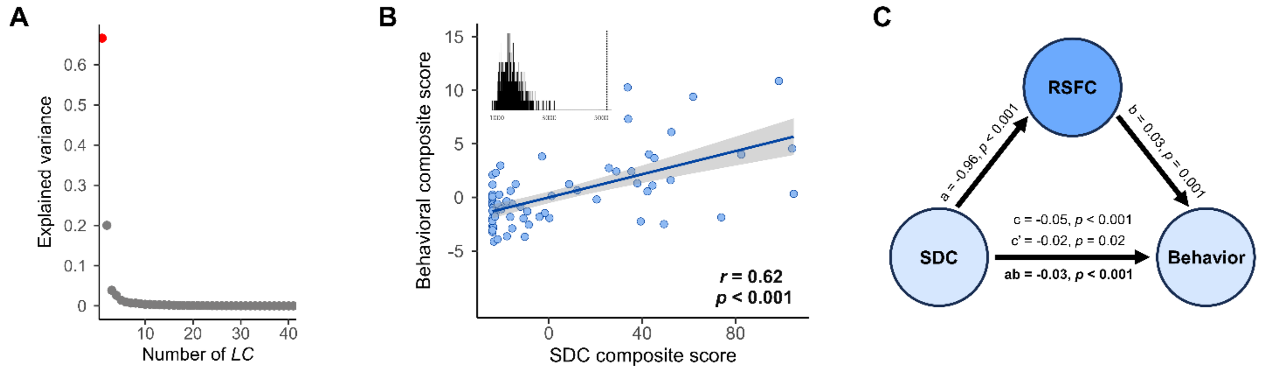

Supplementary Fig. 8. One robust component links structural disconnection with behavioral assessments when the SDC matrix was constructed by an alternative parcellation method. (**A**) The amount of covariance explained by each latent component. The red dot represents the LC that survived after permutation testing with FDR correction ($q < 0.05$). (**B**) Correlations between individual-specific SDC and behavioral composite scores ($p < 0.001$) (n = 81). (**C**) Mediation analysis for SDC composite scores (independent variable), RSFC composite scores (mediating variable) and behavior composite scores (dependent variable). The total effect (c = -0.05, $p < 0.001$) and indirect effect (ab = -0.03, $p < 0.001$) are very significant, and the direct effect (c' = -0.02, $p = 0.02$) is significant.

Supplementary Table 1

Domain	Function tested	Test	Score Recorded
Attention	Average	Posner orienting task, reaction time	Overall performance, RT
	Visual field		Visual field effect [Left-Right], RT
	Shifting		Validity effect [Valid-Invalid], RT
	Shifting		Disengagement effect [(LI-LV)-(RI-RV)], RT
	Average	Posner orienting task, accuracy	Overall performance, accuracy
	Visual field		Visual field effect [Left-Right], accuracy
	Shifting		Validity effect [Valid-Invalid], accuracy
	Shifting		Disengagement effect [(LI-LV)-(RI-RV)], accuracy
	Visual field	BIT star cancellation	Center-of-cancellation, Left vs. Right
		Mesulam unstructured symbol cancellation	Center-of-cancellation, Left vs. Right
Language	Comprehension	BDAE: Comprehension	Basic Word Discrimination
			Commands
			Complex Ideational Material
	Production, semantic	BDAE: Expression	Boston Naming Short Form
	Comprehension	BDAE: Reading	Oral Reading of Sentences
			Comprehension of Oral Reading of Sentences
	Production, phonological	Experimental measures	Nonword Reading
			Stem Completion
	Production, semantic	Verbal fluency	Animal Naming test
Motor	Range of motion	AROM: Shoulder flexion	Left shoulder flexion
			Right shoulder flexion
			Left wrist Extension
			Right wrist Extension
	Strength	Jamar Dynamometer	Left grip strength
			Right grip strength
	Dexterity	Nine-hole peg test	Left hand pegs/second
			Right hand pegs/second
	Dexterity and range of motion	ARAT	Left total
			Right total
	Walking	Timed walk	Index of Timed Walk + FIM Walk Item
		FIM walk item	
	Strength	Motricity Index	Left lower extremity total
			Right lower extremity total
	Range of Motion	AROM: Lower extremity	Left ankle dorsiflexion
			Right ankle dorsiflexion

**Memory**	Spatial, recall	BVMT	Immediate total recall t-score
	Spatial, recognition		Delayed recall t-score
	Verbal, recall	HVLT	Delayed recognition discrimination index
			Immediate total recall t-score
	Verbal, recognition		delayed recall t-score
	Spatial, racall	Spatial span	span forward
			span backward
